# 594 Dermal Matrix versus Autologous Grafting for Full-Thickness Burns of Head and Neck: Trends, Outcomes, Cost

**DOI:** 10.1093/jbcr/irac012.222

**Published:** 2022-03-23

**Authors:** Bianca A Di Chiaro, Kevin E Galicia, Michael J Anstadt, Purvi P Patel, Manuel E Portalatin, Hieu H Ton-That, Richard Gonzalez, Joshua S Carson, John Kubasiak

**Affiliations:** Loyola University Medical Center, Chicago, Illinois; Loyola University Medical Center, Maywood, Illinois; Loyola University Medical Center, Maywood, Illinois; Loyola University Medical Center, Chicago, Illinois; Loyola University Medical Center, Chicago, Illinois; Loyola University Medical Center, Maywood, Illinois; Loyola University Medical Center, Maywood, Illinois; Loyola University Medical Center, Maywood, Illinois; Loyola University Medical Center, Maywood, Illinois

## Abstract

**Introduction:**

Burns of the head and neck are particularly devastating, commonly occurring due to accidental flame or scald injury. Autologous grafting has traditionally been used for reconstruction in this area. Recently the use of dermal matrices as a reconstructive alternative has increased. This study compares annual trends, outcomes, and hospital costs between head and neck burn patients who underwent reconstruction with dermal matrix or autologous grafting.

**Methods:**

The U.S Agency for Healthcare Research and Quality Healthcare Cost and Utilization Project Nationwide Inpatient Sample database was queried from 2012 to 2016. Patients with head and neck burns treated with dermal matrix or autologous graft were identified using International Classification of Disease Ninth and Tenth Revision diagnosis and procedure codes. Demographic, clinical characteristics, and outcome measures were reported. χ ^2^ and Student’s t-tests were used for univariate survey-adjusted comparisons between groups. Multivariate survey-adjusted linear regressions were used to identify associations between surgical intervention, length of stay and cost of hospitalization. Analyses were conducted with RStudio (1.4.1717).

**Results:**

A total of 6,730 patients met inclusion criteria; 2,855 underwent reconstruction dermal matrix and 3,875 with autologous graft. There was an increasing number of patients undergoing dermal matrix reconstruction each year, peak in 2016 (1,450, 50.8%). The dermal matrix group was younger (33.7 vs 39.9, p< 0.001) had fewer females (27.3% vs 38.2%, p=0.003) and fewer inhalation injury (4.0% vs 11.0%, p< 0.001). The dermal matrix group had a shorter length of stay (11.2 vs 27.8, p< 0.001) and more home discharges (80.4% vs 53.4%, p< 0.001); they had lower Elixhauser Comorbidity Index score for readmission (6.57 vs 11.92, p< 0.001) and in hospital mortality (4.76 vs 9.69, p< 0.001). Median cost of care was lower for the dermal matrix group ($17,858 vs $59,860, p< 0.001). Multivariable regression demonstrated that reconstruction with dermal matrix was independently associated with decreased length of stay (11.01 [7.95 - 14.07], p< 0.001) and decreased cost ($38,297 [23,813], p< 0.001).

**Conclusions:**

Operative management of head and neck burns with dermal matrix is associated with a shorter hospital stay, greater likelihood of home discharge, and decreased hospital costs. Patients with autologous grafting had higher rates of inhalation injury, which may account for longer hospital stays and increased discharges to nursing facilities. A limit of this study is the inability to differentiate patients undergoing staged procedures. Further study is needed to evaluate factors contributing to faster discharges home in the head and neck burn population.

